# Membrane lipids from gut microbiome-associated bacteria as structural and signalling molecules

**DOI:** 10.1099/mic.0.001315

**Published:** 2023-03-23

**Authors:** Eileen Ryan, Susan A. Joyce, David J. Clarke

**Affiliations:** ^1^​ APC Microbiome Ireland, University College Cork, Cork, Ireland; ^2^​ School of Biochemistry and Cell Biology, University College Cork, Cork, Ireland; ^3^​ School of Microbiology, University College Cork, Cork, Ireland

**Keywords:** glycine lipids, sphingolipids, plasmalogens, Bacteroidota, inter-kingdom signalling

## Abstract

Bacteria produce an array of diverse, dynamic and often complex lipid structures, some of which function beyond their typical role in membrane structure. The model organism, *E. coli *, has three major membrane lipids, which are glycerophosphoglycerol (phosphatidylglycerol), glycerophosphoethanolamine (phosphatidylethanolamine) and cardiolipin. However, it is now appreciated that some bacteria have the capacity to synthesize a range of lipids, including glycerophosphocholines, glycerophosphoinositols, ‘phosphorous-free’ *N*-acyl amines, sphingolipids and plasmalogens. In recent years, some of these bacterial lipids have emerged as influential contributors to the microbe–host molecular dialogue. This review outlines our current knowledge of bacterial lipid diversity, with a focus on the membrane lipids of microbiome-associated bacteria that have documented roles as signalling molecules.

## Introduction

**Fig. 1.**
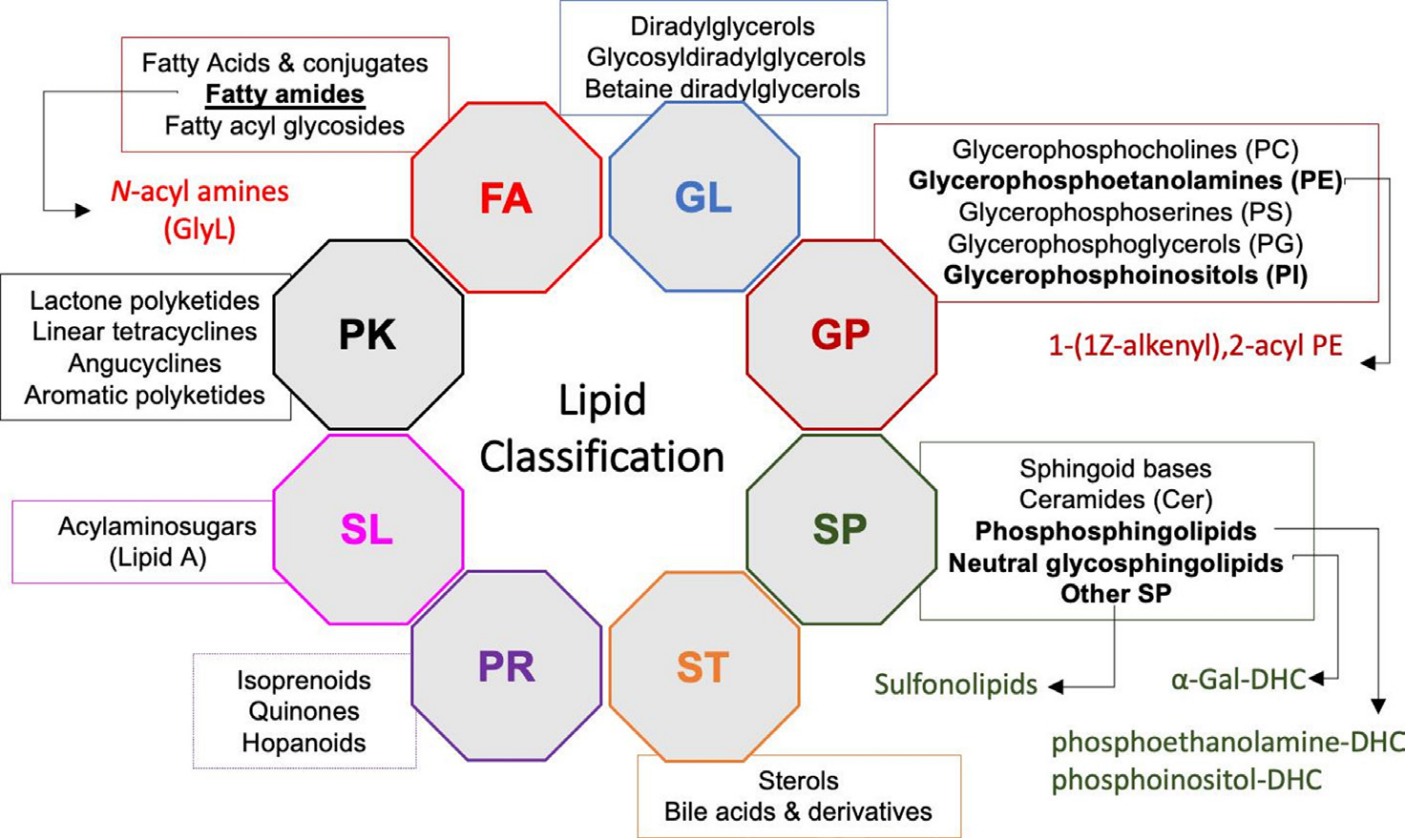
Lipid classification. LipidMaps classify lipids into eight categories (octagons): fatty acyls (FA); glycerolipids (GL); glycerophospholipids (GP); sphingolipids (SP); sterols (ST); prenols (PR); polyketides (PK) and saccharolipids (SL), within which there are distinct classes (rectangles). This is not an exhaustive list of classes but highlights some examples that are most relevant to this review. Within each class of lipids there are also distinct subclasses, with some of the subclasses relevant to microbiome-associated bacteria shown here (with arrows). Adapted from [1–4]. GlyL, glycine lipids; Gal, Galactosyl; DHC, dihydroceramide.

Bacterial membranes contain a pool of diverse hydrophobic or amphipathic lipids that, based on their chemical structures and biosynthetic origins, have been grouped by the LipidMaps consortium into eight categories: fatty acyls (FAs), glycerolipids (GLs), glycerophospholipids (GPs), sphingolipids (SPs), sterol lipids (STs), prenol lipids (PRs), saccharolipids (SLs) and polyketides (PKs), and within each category there are distinct lipid classes and subclasses (see Fig. 1) [[1–4]]. Using LipidMaps nomenclature, the model Gram-negative bacterium, *Escherichia coli,* has a membrane that is composed of three lipids from the GP category, glycerophosphoglycerol (phosphatidylglycerol; PG), glycerophosphoethanolamine (phosphatidylethanolamine; PE) and cardiolipin (CL) and the biosynthesis of these lipids has been well described in previous reviews [5–7]. However, it is now appreciated that some bacteria have the capacity to synthesize additional GPs, such as glycerophosphocholines (phosphatidylcholines; PCs) and glycerophosphoinoistols (phosphatidylinositols; PIs) [5, 8, 9]. Bacterial membranes can also contain phosphate-free lipids that may be produced during periods of phosphate starvation, presumably to replace the phosphate-rich GPs in the bacterial membranes [5, 10–12]. For example, *N*-acyl amines are acylated amino acids that are produced by many Gram-negative bacteria. *N*-acyl amines do not contain any phosphorous and may be mono- or di-acylated and may involve one (ornithine, lysine, glycine, or glutamine) or more (glycine-serine, ornithine-taurine, glycine-serine-ornithine) amino acids [10, 11, 13–22]. Sphingolipids (SPs) and the structurally related sulfonolipids are important membrane lipids produced by members of the phylum Bacteroidota, including genera such as *Bacteroides* and *Odoribacter* that are associated with the human gut microbiome [5, 23–27]. Whilst relatively rare, the production of STs, a class of tetracyclic triterpenoid lipids, by diverse bacteria has been reported [28–31]. It is believed that approximately 10 % of bacteria have the appropriate genes to produce the prenolic hopanoids (Fig. 1), which, like STs, are planar polycyclic hydrocarbons containing five phenolic rings rather than the four rings found in STs [32].

Therefore, there does appear to be a large diversity of lipids present in bacteria and, although many of these lipids are likely to have an important structural role in bacterial membranes, a subset of bacterial lipids produced by members of the human gut microbiome have emerged as ligands for host receptors and may make a significant contribution to the host–microbe dialogue. These signalling lipids include specific GP species, *N*-acyl amines and SPs from both gut and oral-associated members of the human gut microbiota. This review details the diversity, biosynthesis and function of these new lipid ligands, as well as other bioactive membrane lipids from gut microbiota-associated bacteria such as sulfonolipids and plasmalogens.

## GPs as signalling molecules in the microbe–host dialogue

**Fig. 2.**
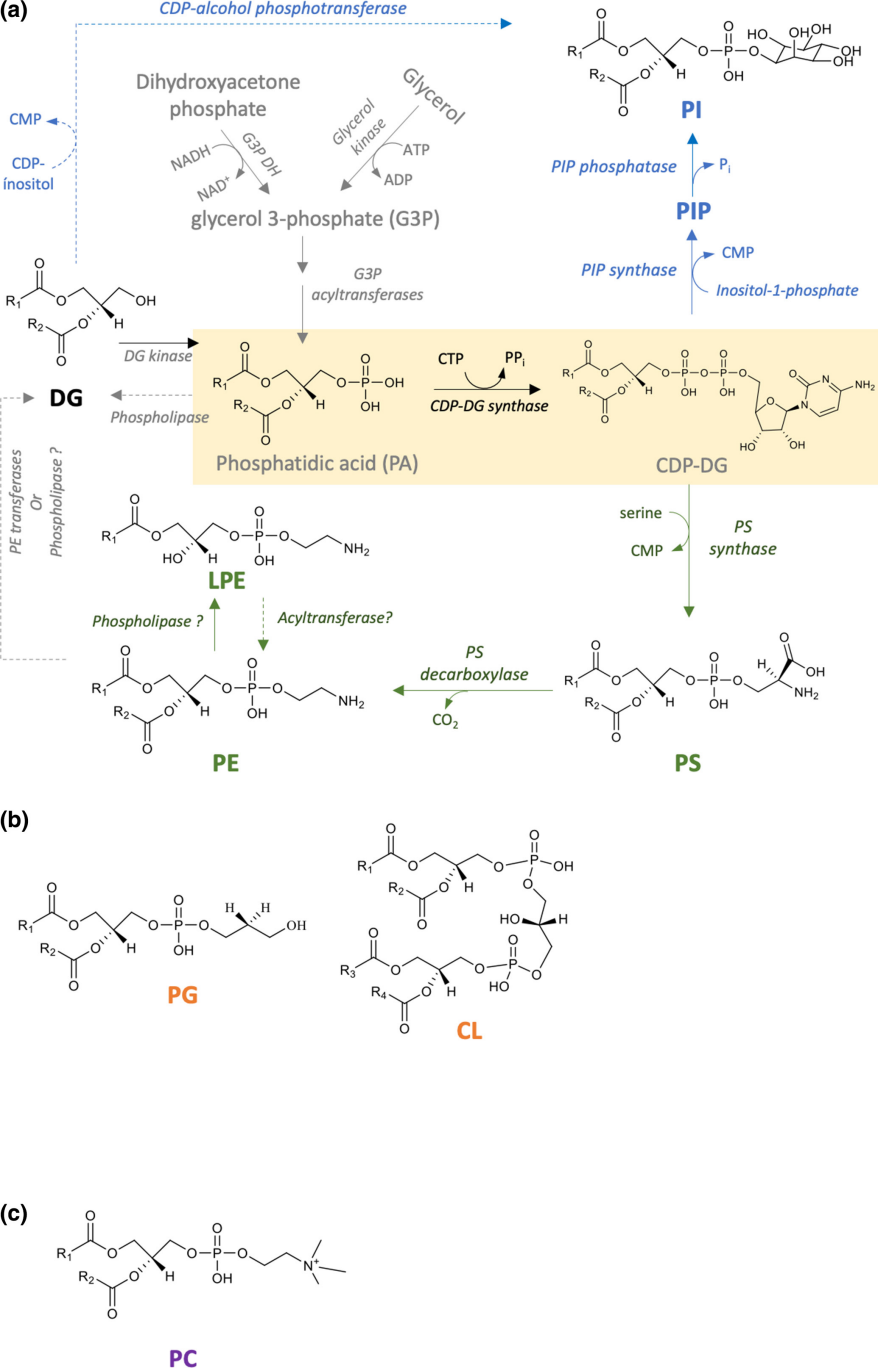
Biosynthesis of glycerophophoethanolamine (PE) and glycerophophoinoistol (PI) in bacteria. (**a**) In bacteria glycerol-3-phosphate (G3P) forms the backbone of all glycerophospholipids (GPs). Subsequent di-acylation of G3P leads to the formation of phosphatidic acid (PA), which is then converted to cytidine diphosphate-diacylglycerol (CDP-DG). PA may also be formed by the phosphorylation and recycling of DG, a metabolic by-product generated from the degradation of GPs. The biosynthesis of PE and PI starts from the central metabolite CDP-DG, leading to the production of either gylcerophosphoserine (phosphatidylserine, PS) or PI. The latter is formed via PI phosphate (PIP) intermediates, or there is some evidence that PI can be formed directly via diacylglycerol (DG) and CDP-alcohol-phosphotransferase (see text for further details). PS is produced from two substrates, CDP-DG and serine, via PS synthase. Thereafter, PE is typically synthesized via the decarboxylation of PS. Mono-acylated or lyso PE (LPE) are generated as metabolic intermediates in phospholipid synthesis or from membrane degradation via the action of phospholipases. (**b**) The structures of glycerophosphoglycerol (phosphatidylglycerol, PG) and cardiolipin (CL), which are major membrane lipids of many bacteria, including *E. coli *. (**c**) The structure of glycerophosphocholine (phosphatidylcholine, PC).

GPs are characterized by the presence of a phosphate (or phosphonate) group, which in eukaryotes and eubacteria is esterified to the hydroxyl group at the sn-3, and in archaea, to the sn-1 position of glycerol. A variety of GPs with different headgroups are synthesized by different bacteria (Fig. 2); the charges of zwitterionic GPs (PE, PC) must be balanced with those containing acidic headgroups (PG, CL, PS, PI) so that some integral membrane proteins can adopt the correct topology in the cell membrane [33]. As with many Gram-negative bacteria, PE is also a major lipid in the gut microbiome-associated Bacteroidota [6]. PS has been detected as a minor lipid in several species of *Bacteroides *, indicating a likely role as an intermediate to PE biosynthesis in this genus (see Fig. 2) [34]. Beyond the membrane, some species of PE appear to be important in microbiome–host interactions and an immunomodulatory role was very recently described for a specific PE isolated from the human symbiont, *Akkermansia muciniphila* [35]. Numerous studies have shown that *A. muciniphila* plays a major role in regulating human immune responses in a variety of contexts [36]. It was demonstrated that this immunomodulatory activity can be replicated by a specific PE that contains 2 saturated fatty acyl chains with 15 carbons, 1 with a terminal anteiso (a) branched methyl group and the other with an iso (i) branch, i.e. PE a15 : 0-i15 : 0 [35]. The immunogenic activity of this PE species requires a TLR1–TLR2 heterodimer and is remarkably specific, for example methyl branches are essential for activity and the two acyl chains must be different (i.e. anteiso and iso branched not both anteiso or both iso) [35]. In bacteria, PE tends to be present in the diacylated form, with mono-acylated (lyso) PE typically accounting for only a minor fraction (≤1 %) of the bacterial membrane [34, 37]. It is generally thought that lyso GPs are generated as metabolic intermediates in GP synthesis or from membrane degradation via the action of endogenous phospholipase A2 and, as inverted cone-shaped molecules, lyso GPs share physical characteristics of detergents, enabling modification of local membrane properties such as curvature [37]. More specifically, it has been mentioned that lyso PEs isolated from *Chitinophaga* have antimicrobial activities against certain Gram-positive bacteria [21], whilst short-chain lyso PEs from *Campylobacter jejuni* were shown to be toxic to host cells and thus they may be considered as a novel virulence factor of this bacterium [38].

It has very recently emerged that PI, a membrane GP typically produced by the Actinobacteria (including *Mycobacterium *, *Corynebacteria* and *Streptomyces *), is also widespread among host-associated members of the Bacteroidota [5, 9]. The loss of inositol lipid production in *Bacteroides thetaiotaomicron* resulted in loss of both capsule expression and increased antimicrobial peptide resistance, *in vitro*, whilst loss of inositol lipids also decreased bacterial fitness in a gnotobiotic mouse model [9]. In all domains of life, PI biosynthesis begins with the generation of inositol-phosphate from glucose-6-phosphate via myo-inositol-phosphate synthase (MIPS) [39]. From here, PI biosynthesis can occur using two different routes (see Fig. 2).

The first route, which is typical of *B. thetaiotaomicron *, involves the synthesis of a phosphoinositol phosphate (PIP) intermediate by the reaction of inositol phosphate with CDP-DG via PIP synthase and subsequent dephosphorylation of PIP to PI via PIP phosphatase [5, 9]. An alternative route for PI biosynthesis, predicted *in silico* but not yet fully characterized, appears to be present in some Bacteroidota, and this route lacks a PIP intermediate and resembles instead the eukaryotic Kennedy pathway for PE synthesis [9]. In this route, CDP-inositol is first generated from the reaction of inositol phosphate and CTP via a nucleotidyltransferase (NTP) and thereafter CDP-inositol and diacylglycerol (DG) are converted to PI via a CDP-alcohol phosphatidyltransferase [9] (Fig. 2). The generation of CDP-inositol to be used as a substrate for bacterial phospholipid synthesis has been previously reported in hyperthermophiles [40]. Therefore, host-associated Bacteroidota appear to encode either a PIP-dependent or a PIP-independent pathway for the biosynthesis of PI, although there are also notable exceptions where the genetic capacity for PI synthesis appears to be absent or infrequent, e.g. *Bacteroides fragilis *. Interestingly, phosphorylated derivatives of PI (phosphoinositides, PtdIns) have a very important signalling role in eukaryotes with significant impacts on all aspects of cell physiology [41]. It will be interesting to determine if bacterial-derived PI can contribute to this signalling in any way.

## Plasmalogens are widespread in anaerobic bacteria, including certain gut-associated *Bacteroides*

**Fig. 3.**
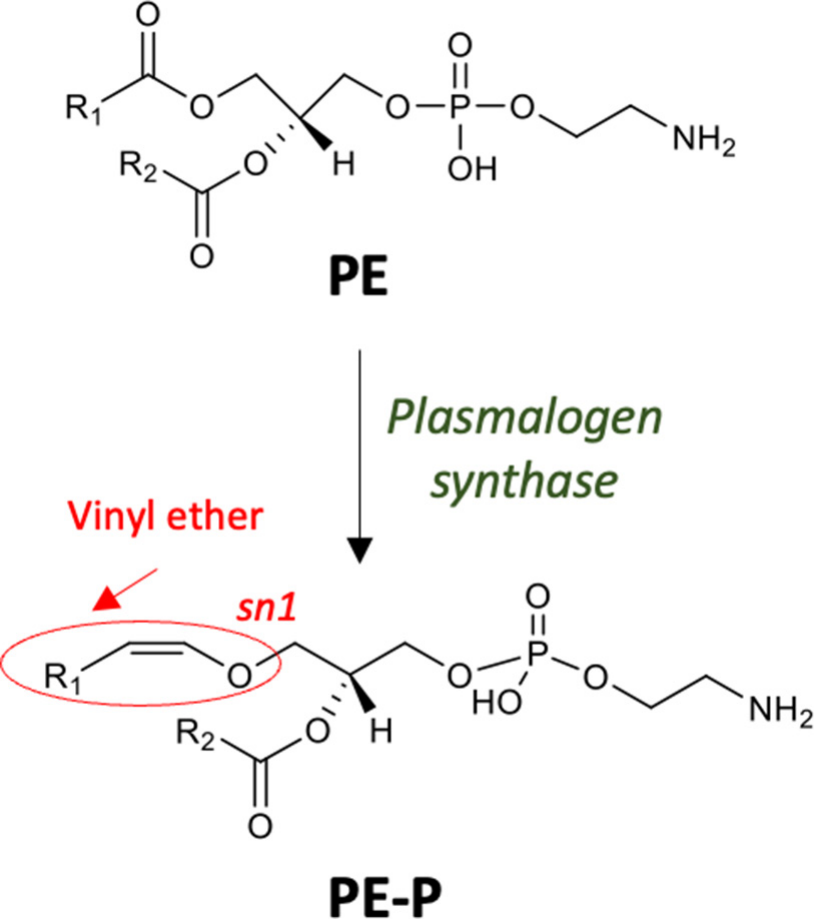
Biosynthesis of plasmalogens in anaerobic bacteria. The biosynthesis of plasmalogens is mediated by the reduction of glycerophospholipids such as PE to 1-(1Z-alkenyl),2-acyl PE (PE-P) through the activity of plasmalogen synthase [49].

Plasmalogens, or vinyl ether lipids, are produced by the modification of the fatty acid at the sn-1 position of GPs such that it is linked via an alkenyl or plasmenyl bond rather than an ester bond (Fig. 3). Plasmalogens have a broad phylogenetic distribution, being found in many biological membranes from bacteria, protozoa, invertebrates and mammals. Amongst bacteria, with the exception of myxobacteria [42], plasmalogens are not found in aerobes and are rarely found in facultative anaerobes, but appear to be common in anaerobes, including certain gut-associated *Bifidobacteria*, *Clostridia* and *Bacteroides* [34, 43–49]. In mammals, plasmalogen biosynthesis involves the oxygen-dependent conversion of an ester to a vinyl ether [50, 51]. Oxygen, therefore, has a prominent role in the production of plasmalogens in eukaryotes, and it was not immediately clear how anaerobic bacteria could produce these lipids. However, recently it has been shown that proteins with homology to enzymes in the benzoyl-CoA reductase (BCR) and 2-hydroxylacyl-CoA dehydratase (HAD) family are responsible for plasmalogen biosynthesis in anaerobic bacteria [49]. Specifically, a two-gene operon in *Clostridium perfringens *, *plsAR*, that was required for the conversion of GPs (such as PE) to their corresponding plasmalogen, was identified [49]. The PlsA and PlsR proteins contain activation and reduction/dehydration domains that exploit the reductive power associated with electron transfer to generate the vinyl ether bond. Homologues of PlsA and PlsR have been identified in many anaerobic bacteria, including ~15 % of the sequenced Bacteroidota [49].

In mammals, plasmalogens play unique roles in membrane structure, membrane trafficking and cell signalling, with reduced levels of circulating plasmalogens linked to several metabolic and neurological diseases, including diabetes and Alzheimer’s [52–54]. The exact roles of plasmalogens in bacteria are not known, but, by virtue of their vinyl ether bond, they are likely to be involved in the modulation of membrane morphology and protection from oxidative stress [55]. It is worth noting that the ability to synthesize plasmalogens can be sporadic across a genus, indicating that these lipids are not essential but may have an adaptive and/or fitness role in bacteria. For example, plasmalogens derived from PG and CL were detected in *Bifidobacterium longum *, but not in *Bifidobacterium animalis* [45], whilst PE plasmalogens were detected in *B. thetaiotaomicron* but not in *B. fragilis *, *B. vulgatus* or *Bacteroides ovatus* [34].

## N-acyl amines produced by members of the Bacteroidota function as GPCR and TLR ligands

**Fig. 4.**
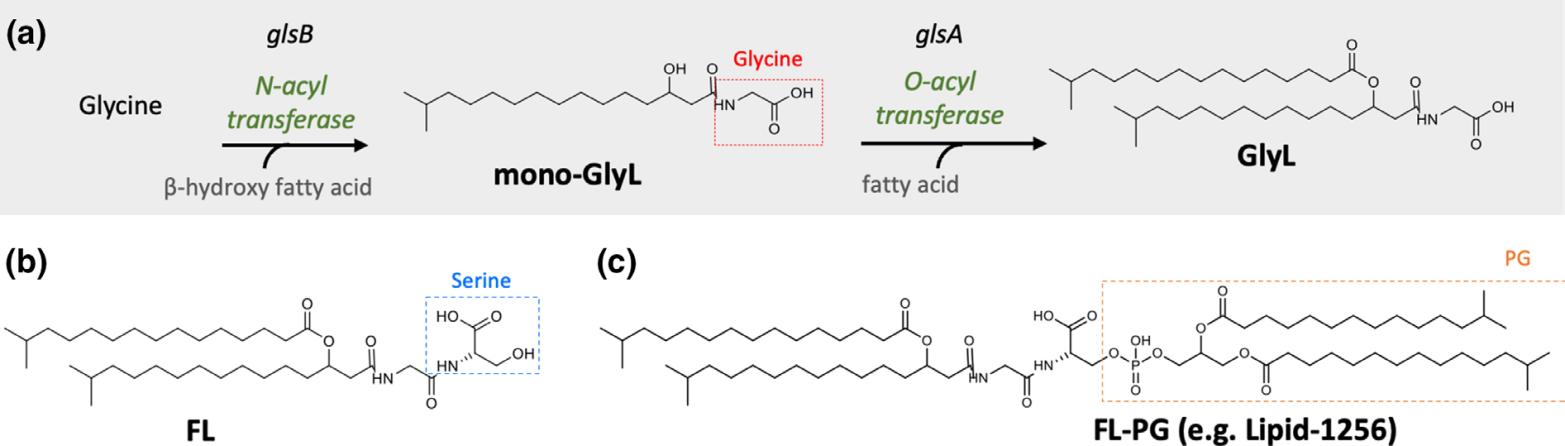
Biosynthesis of glycine lipids in *Bacteroidetes *. (**a**) The first step involves *N*-acylation of glycine with a primary β-hydroxy fatty acid via *N*-acyltransferase (encoded by *glsB*), generating a mono-acylated amine (mono-GlyL). This is followed by subsequent *O*-acylation (esterification) of mono-GlyL with a secondary fatty acid via *O*-acyltransferase (encoded by *glsA*) to produce a mature diacylated glycine lipid, GlyL [11, 57]. (**b**) The exact pathway for the biosynthesis of the related glycine-serine dipeptide lipids known as flavolipins (FLs) is unknown but hypothetically they could be synthesized from the respective glycine lipid precursors by attaching serine to the terminal glycine moiety [58]. (**c**) Complex chimeric GlyL-derived molecules such as lipid 1256 have also been detected in *Bacteroides* and *Porphyromonas *, although the exact biosynthetic route for these complex lipids is also unknown (see text for further details).

Glycine lipids (GlyLs) were first described as cytolipin in the gliding bacterium *Cytophaga johnsonae* [14]. Since then, GlyLs and the related glycine-serine dipeptido-lipids (flavolipins, FLs) (Fig. 4a, b) have been identified in several different members of the phylum Bacteroidota, including those associated with the gut and oral microbiomes [11, 14, 15, 18, 21, 34, 56–59]. A range of mono- and di-acylated GlyL and FL species, varying in acyl chain length, have been detected in different *Bacteroides* species, as well as the oral pathogen *Porphyromonas gingivalis* [34, 57, 60]. In *B. thetaiotaomicron *, GlyLs have been shown to be important for resistance to bile salts and colonizing the mammalian gut [57]. GlyL biosynthesis in *B. thetaiotaomicron* begins with the *N*-acylation of glycine with a primary β-hydroxy fatty acid via *N*-acyltransferase activity encoded by the *glsB* gene, generating a mono-acylated amine, such as *N*-acyl-3-hydroxy-palmitoyl glycine (commendamide) (Fig. 4a). This is followed by subsequent *O*-acylation (esterification) of the acylated molecule with a secondary fatty acid via *O*-acyltransferase activity (encoded by *glsA*) to produce a mature diacylated amino acid lipid (Fig. 4a) [57].

More complex GlyL, e.g. lipid 1256, which consists of PG or PE linked to FLs (see Fig. 4c), have also been observed [34, 57, 60]. Notably, analysis of MS data (based on *m*/*z* values) suggests the presence of a FL–PE complex in *Bacteroides *, in contrast to the FL–PG complex identified in *P. gingivalis* [34, 58]. Although the exact route of biosynthesis for lipid 1256 and FLs has not been confirmed, these lipids are absent from the *glsB* mutant of *B. thetaiotaomicron *, indicating that they are derived, at least in part, from GlyL biosynthesis (see Fig. 4) [34, 58].

GlyLs have been detected in the serum and brain of healthy individuals, indicating that these microbial lipids are distributed around the human host [56]. A recent study has reported the presence of GlyLs in outer-membrane vesicles (OMVs) produced by *B. thetaiotaomicron *, indicating that OMVs may facilitate the systemic distribution of these lipids [61]. GlyLs have also been reported to activate several signalling pathways in the host. Mono-acylated GlyLs produced by commensal bacteria activate G-protein-coupled receptors (GPCRs) in the host and may function similarly to endogenously produced endocannabinoid-like *N*-acyl ethanolamines [62]. Specifically, commendamide has been shown to activate GPCR G2A/132, resulting in increased levels of NF-kB expression [62, 63].

Interestingly, gut-inhabiting members of the Clostridia such as *Eubacterium rectale* also produce *N*-acyl amines [called fatty acid amines (FAAs)] that can function as GPCR agonists [64]. Moreover, it has been reported that some FAAs produced by the gut microbiota can influence host behaviour by increasing motivation to do exercise and this behaviour was shown to be mediated through the FAA-dependent activation of the endocannabinoid receptor CB1 and the subsequent stimulation of TRPV1-sensory neurons in the periphery of the host [65]. A very recent study has identified *A. muciniphila* as a major source of another group of *N*-acyl amines called ornithine lipids (OLs) in the gut [66]. OLs are well characterized in free-living bacteria and these lipids resemble GlyLs except that glycine has been replaced with the amino acid ornithine [11]. In the gut, OLs appear to be immunomodulatory and therefore potentially have an important role in mediating *Akkermansia *–host interactions [66].

GlyLs (including FLs) isolated from *P. gingivalis* are reported to signal to eukaryotic cells by engaging TLR2, promoting the production of pro-inflammatory cytokines [56, 60, 67]. More recently, lipid 1256 was shown to be an even more potent TLR2 agonist [58]. Lipid 1256 has also been detected in diseased dental tissues in greater amounts than healthy tissue and is purported to drive inflammation and localized dental tissue destruction [59]. Interestingly an ‘anti-inflammatory’ effect of FLs has also been reported whereby chronic (7 week) intraperitoneal administration of FLs to high-fat-diet (HFD)-fed low-density lipoprotein receptor (Ldlr−/−) mice resulted in lowered cholesterol, attenuated atherosclerosis progression and decreased markers of liver injury compared with vehicle control-injected mice [68].

## Sphingolipids (SPs) are essential lipids of host-associated *Bacteroidetes* with roles in persistence, homeostasis and inflammation

**Fig. 5.**
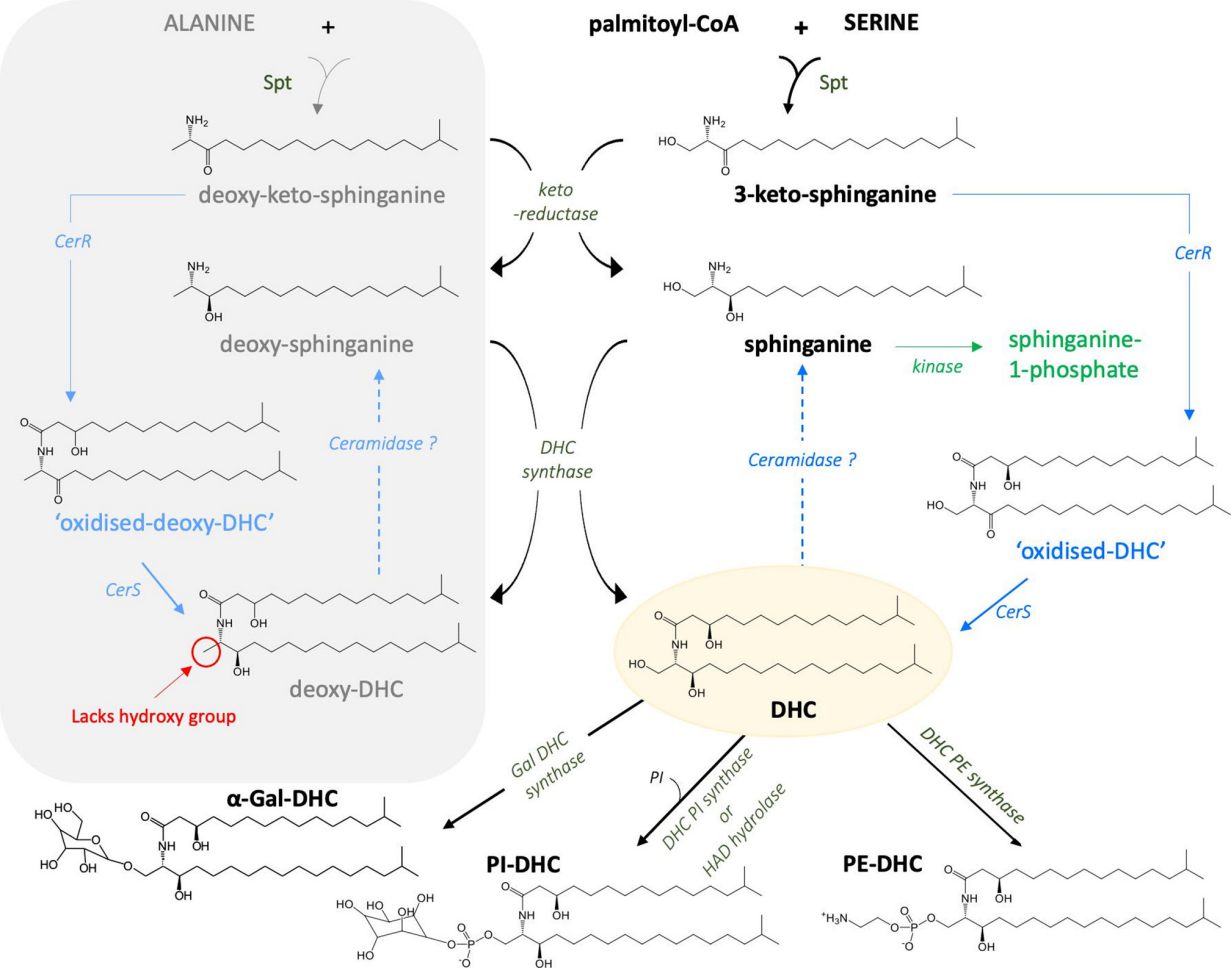
Biosynthesis of SP in the Bacteroidota. The biosynthesis of SPs is initiated by serine palmitoyltransferase (Spt), which catalyses a reaction between palmitoyl-CoA and serine to form 3-ketosphinganine. Thereafter, the pathway to dihydroceramide (DHC) synthesis may proceed via 3-ketosphinganine reductase activity to form sphinganine or alternatively bacterial Cer synthase (CerS) directly adds an acyl chain to 3-keto-sphinganine, producing an ‘oxidized-DHC’ intermediate that is then reduced to DHC by bacterial Cer reductase (CerR). DHC represents the central hub of SP metabolism and undergoes modification with different head groups to produce the uniquely bacterial SPs, i.e. PE-DHC, PI-DHC and α-Gal-DHC. Sphinganine may be directed away from the synthesis of DHC via the formation of sphinganine-1-phosphate via a novel kinase recently discovered in *P. gingivalis* [105]. Spt can also catalyse the condensation of alternative amino acids (such as alanine) with palmitoyl-CoA, resulting in the formation of deoxy-DHC, a metabolite that lacks the hydroxyl group required for the addition of the various head groups. Therefore deoxy-DHC can accumulate to toxic levels in the cell. Note: structures are based around C17 sphinganine backbone (branched) and hydroxy C17 fatty acid (branched).

SPs are characterized by a long-chain amino alcohol sphingoid backbone with an amide bound fatty acyl chain (see Fig. 5). Structural diversity arises through variation in the lipid headgroup (simple or branched sugar residues, or neutral or charged moieties) and sphingoid base/fatty acyl chain (length, degree of saturation, methylation or branching that is typical of bacterial SPs not eukaryotic) [24]. SPs are ubiquitous membrane lipids in eukaryotes that participate in the generation of various membrane structures, including rafts, caveolae and cytosolic vesicles, but their detection in bacteria is largely limited to members of the Bacteroidota and certain *α-*Proteobacteria, e.g. *Caulobacter crescentus* [23, 69]. The biosynthesis of all SPs is dependent on the initial and rate-limiting step that requires serine palmitoyl transferase (Spt), an enzyme that catalyses the condensation of serine and palmitoyl-CoA, resulting in the formation of 3-ketosphinganine [24, 70, 71]. In eukaryotes the next step in SP production is the reduction of 3-ketosphinganine to sphinganine through the action of a 3-ketosphinganine reductase [72, 73]. Sphinganine can be detected in *Bacteroides* and a gene encoding a protein with 3-ketosphinganine reductase activity (BT_0972) has recently been identified in *B. thetaiotaomicron* [74]. Moreover, dietary sphinganine can be selectively assimilated by *Bacteroides* and converted to more complex SPs [75]. Therefore there is evidence for a SP biosynthetic pathway in *Bacteroides* that resembles the canonical pathway described in eukaryotes. However, one important distinction is that bacteria produce dihydroceramide (DHC) as distinct from ceramide in eukaryotes. Recent work in *Caulobacter* has suggested that a second SP biosynthesis pathway may be present in bacteria whereby 3-ketosphinganine is first converted to ceramide through the action of a bCerS and this is followed by a reduction [via ceramide reductase (bCerR)], resulting in the production of DHC (see Fig. 5) [76]. Interestingly, potential homologues of bCerR have also been identified in *Bacteroides *, suggesting that there may be at least two separate pathways for DHC biosynthesis in these gut bacteria [76].

In bacteria, DHC is modified by the addition of different headgroups to produce a range of SPs. For example, a recent comparative study of four members of the genus *Bacteroides* identified three major SPs, i.e. α-galactosyl-dihydroceramide (α-Gal-DHC), dihydroceramide phosphoethanolamine (PE-DHC) and dihydroceramide phosphoinositol (PI-DHC) [34]. PE-DHC is produced by all *Bacteroides* species tested but the production of α-Gal-DHC and PI-DHC does appear to be species-specific [34]. The biosynthesis of PI-DHC involves either PI-DHC synthase (typical of *B. thetaiotaomicron *) or haloalkanoate dehalogenase (HAD) hydrolase activity (typical of *B. vulgatus *) [9]. *B. fragilis* α-Gal-DHC was recently reported to be formed via a ceramide UDP-GalCer synthase [77].

Spt can also acylate non-cognate amino acids (such as alanine or glycine), resulting in the production of deoxysphingolipids (deoxy-SP) [71]. In eukaryotes deoxy-SP are toxic metabolites that have been implicated in neurological and metabolic disorders [71]. We, and others, have detected deoxy-DHC in *Bacteroides* and, therefore, the production of this toxic metabolite by members of the gut microbiota could have consequences in the host (see Fig. 5) [25, 34].

SP production is required for a normal response to stress and persistence in both *Bacteroides* and *P. gingivalis* [78, 79]. Moreover, it has been reported that the synthesis of SPs is critical to the presentation of surface polysaccharides in *P. gingivalis* [79]. This might explain why human THP1 macrophage-like cells exposed to *P. gingivalis* defective in SP production elicit a hyper-inflammatory response via elevated IL-1β, IL-6, IL-10, IL-8, RANTES and TNFα when compared to THP-1 cells exposed to wild-type bacteria [80].

The application of a click chemistry approach [BOSSS (BioOrthogonal-Sort-Seq-Spec)] has clearly shown that bacterial SPs produced in the gut can be distributed to distal sites in the host and contribute to the host ceramide pool [81, 82]. A recent study identified a unique homoserine-DHC that was produced by *B. thetaiotaomicron* in the gut of mice and, using BOSSS, this lipid was shown to transfer to colon and liver tissue. In addition, the homoserine-DHC induced transcriptional changes in cultured HepG2 cells that impacted on oxidative respiration and fat accumulation [82]. Interestingly, these authors also reported that diet-induced hepatic steatosis in mice could be improved by oral gavage of wild-type *B. thetaiotaomicron *, but this improvement was not observed following gavage with a mutant unable to produce SPs [82]. The mammalian host also appears to compensate for the absence of bacterial SP production in the gut by increasing production of endogenous SPs, resulting in inflammation in the gut [25]. Although the mechanism(s) used for the delivery of bacterial SPs to the host is not known, it is likely to involve OMVs [81]. Specific bacterial SPs have been shown to have important inter-kingdom signalling functions. For example, α-Gal-DHC produced by *B. fragilis* binds to the antigen-presenting molecule CD1d, influencing the number and function of natural killer T cells (NKT-cells) in the intestine and the progression of a murine model of colitis [83, 84]. The bioactive α-Gal-DHC was shown to consist of a C18 sphinganine backbone and a hydroxy C16 : 0 fatty acyl chain, and this was distinct from the prominent α-Gal-DHC reported to consist of C17 sphinganine backbone and a hydroxy C17 : 0 fatty acyl chain [84, 85]. Therefore bacterial SP can impact on host physiology through either a general contribution to the host SP pool or through specific ligand–receptor interactions. With this mind, it is worth pointing out that each person is expected to have a unique combination of SP-producing bacteria in their gut and therefore a better understanding of the full extent of the signalling potential of different bacterial SP species is critical for a more complete appreciation of the role of bacterial SPs in the host–microbe dialogue.

## Sulfonolipids: unique and bioactive sphingolipids of *Bacteroidota*

Sulfonolipids can be described as an unusual class of SP containing a sulfonic acid group in the sphingoid base (rather than serine). Synthesis of sulfonolipids in bacteria appears to be limited to the phylum Bacteroidota and may be mutually exclusive to SPs, i.e. members of the Bacteroidota have the capacity to produce either SPs or sulfonolipids. Sulfonolipids are abundant membrane constituents of bacteria from the *Cytophagia–Flavobacteria* group [86–91]. Sulfonolipids are also characteristic of the genus *Capnocytophaga* (residents of the oral microbiome), gut-associated *Alistipes* and *Odoribacter *, and, very recently, sulfonolipids have been isolated from the opportunistic pathogen *Chryseobacterium gleum* [26, 27, 86, 92].

**Fig. 6.**
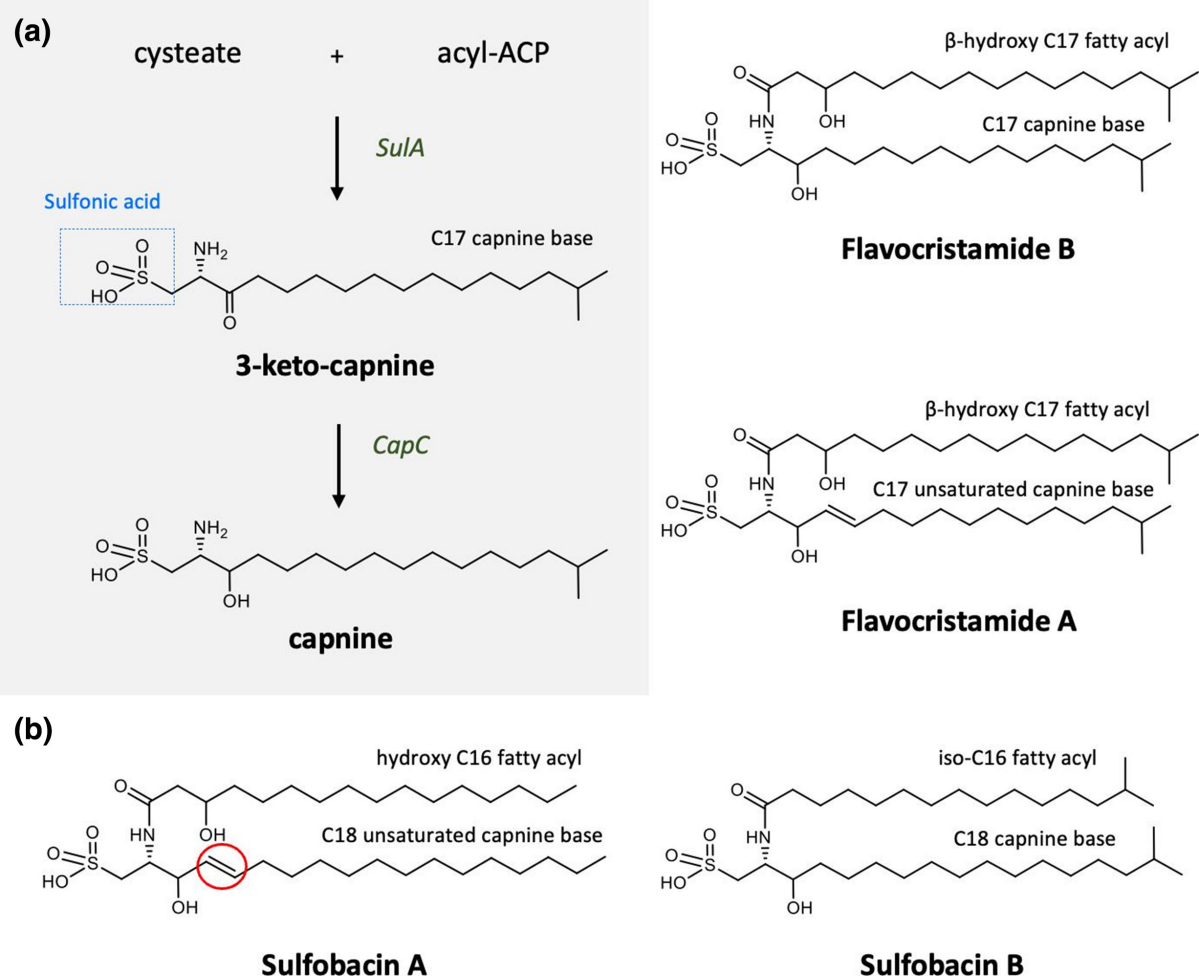
Biosynthesis of sulfonolipids. (**a**) The biosynthesis of sulfonolipids is initiated by the condensation of cysteate and acyl-acyl carrier protein (ACP) to form 3-keto capnine via an acyl-ACP transferase enzyme (SulA). Keto-capnine is then reduced to capnine via a 3-keto capnine reductase (CapC) (see text for further details)). (**b**) Thereafter, the *N*-acylation of capnine with different fatty acids (hydroxy or non-hydroxy) leads to the generation of varying sulfonolipids, such as sulfobacins (C18 capnine base) and flavocristamides (C17 capnine base).

A recent study in *Capnocytophaga* identified three proteins involved in the production of sulfonolipids [93]. CapA is required for the production of cysteate through the sulfonation of phosphoserine and CapB catalyses the condensation of cysteate with a fatty acyl-ACP, resulting in the generation of 3-ketocapnine that is subsequently reduced to capnine via a 3-ketocapnine reductase called CapC [93] (see Fig. 6). Thereafter, *N-*acylation of capnine with different iso-fatty acids leads to the generation of different sulfonolipids such as sulfobacin A, sulfobacin B and flavocristamide A [26]. Another recently published study identified a protein called SulA as the CapB homologue in the human gut-associated bacterium *Alistipes finegoldii* [94]. Interestingly, *Alistipes* – and *Odoribacter* – do not produce cysteate using a homologue of CapA, but rather these bacteria have a homologue of the distantly related archaeal cysteate synthase, suggesting a phylogenetic split in how environmental and gut-associated Bacteroidota produce the cysteate required for sulfonolipid production [94].

Sulfonolipids have been shown to be required for gliding motility [95–97]. Moreover, sulfonolipids can be bioactive molecules and sulfobacins A and B are known to be antagonists of the von Willebrand receptor involved in platelet aggregation and thrombosis [98]. Similar sulfonolipids termed flavocristamides A and B, isolated from a marine bacterium *Flavobacterium* sp. and the Gram-negative seawater bacterium *Cyclobacterium marinus *, have been shown to inhibit DNA polymerase [99]. The rosette-inducing factor (RIF-1) produced by *Algoriphagus machipongonensis* plays an important role in regulating the formation of multicellular rosettes by the choanoflagellate *Salpingoeca rosetta* [89]. Therefore sulfonolipids are involved in regulating the transition from a single-celled animal to a multi-cellular animal. On the other hand, sulfonolipids have been reported to display cytotoxic and pro-inflammatory activities [92, 100]. For example, sulfobacin A, isolated from *Chryseobacterium gleum* was shown to exhibit pro-inflammatory activity in mice primary macrophages via an increased expression levels of several pro-inflammatory cytokines including IL-1α, IL-1ß, IL-6 and TNFα, indicating a potential role of sulfonolipids in pathogenicity [92].

## Conclusion and future perspectives

Lipids are important molecules that are essential for membrane structure and function in all cells. However, our knowledge of the full functional range of bacterial lipids is still very limited. In this review we have focused on describing recent advances in our understanding of the structure and role of some of the novel lipids produced by members of the human gut microbiota, such as *Bacteroides *. In addition to the typical phospholipids (in the GP category) found in bacterial membranes, *Bacteroides* and other members of the Bacteroidota have been shown to produce several additional lipids, including SP and GlyL [24, 57, 58]. Both SP and GlyL have been shown to be important structural components of the *Bacteroides* Gram-negative cell wall [25, 57, 78, 79]. Membrane homeostasis (including OM asymmetry) and membrane lipid transport (particularly GPs) in Gram-negative bacteria have been well-studied in *E. coli* [101, 102]. Are the same mechanisms used to transport GlyLs and SPs in *Bacteroides* or do novel lipid transport systems remain to be identified? Both bacterial SPs and GlyLs have been shown to interact with host signalling proteins and have been detected in sites distal to the gut, such as the liver and the brain [24, 56, 58, 60, 81–84, 103]. Therefore, these lipids have the potential to act as systemic inter-kingdom signalling molecules. However, what is the full repertoire of lipids produced by the Bacteroidota and other (gut-associated) bacteria and how do these lipids interact with the host? For example, what is the role of plasmalogens in *Bacteroides *? In humans, plasmalogens can act as a carrier for PUFA (typically linked to sn2 position) that are the precursors to lipid mediators (oxylipins) involved in inflammation (pro-inflammation and pro-resolving) [53]. Therefore, can bacterial plasmalogens act as carriers for lipid mediators in the host? It is also possible that bacterial plasmalogens might contribute to the plasmalogen pool in the host (as has been shown for bacterial SPs) and this could provide new therapeutic avenues for conditions associated with plasmalogen deficiency in humans [104]. Finally, why does the production of sulfonolipids and SPs appear to be mutually exclusive in the Bacteroidota? What are the evolutionary pressures that select for SPs rather than sulfonolipids? Interestingly, the gut-associated Bacteroidota appear to favour the production of SPs over sulfonolipids (with the exception of *Alistipes* and *Odoribacter *), suggesting that there might be an adaptive advantage for production of SPs in the mammalian gut environment. Characterizing the full signalling potential of these, and yet to be described, lipids is therefore critical for a more complete understanding of the molecular dialogue between animals and their microbiota.
